# Otoacoustic Emissions Evoked by the Time-Varying Harmonic Structure of Speech

**DOI:** 10.1523/ENEURO.0428-20.2021

**Published:** 2021-04-08

**Authors:** Marina Saiz-Alía, Peter Miller, Tobias Reichenbach

**Affiliations:** Department of Bioengineering and Centre for Neurotechnology, Imperial College London, London SW7 2AZ, United Kingdom

## Abstract

The human auditory system is exceptional at comprehending an individual speaker even in complex acoustic environments. Because the inner ear, or cochlea, possesses an active mechanism that can be controlled by subsequent neural processing centers through descending nerve fibers, it may already contribute to speech processing. The cochlear activity can be assessed by recording otoacoustic emissions (OAEs), but employing these emissions to assess speech processing in the cochlea is obstructed by the complexity of natural speech. Here, we develop a novel methodology to measure OAEs that are related to the time-varying harmonic structure of speech [speech-distortion-product OAEs (DPOAEs)]. We then employ the method to investigate the effect of selective attention on the speech-DPOAEs. We provide tentative evidence that the speech-DPOAEs are larger when the corresponding speech signal is attended than when it is ignored. Our development of speech-DPOAEs opens up a path to further investigations of the contribution of the cochlea to the processing of complex real-world signals.

## Significance Statement

Real-world environments, such as a loud pub or restaurant, are often noisy. The detection of sound occurs in the inner ear, which also possesses an active mechanism to mechanically amplify sound vibrations. Because the active mechanism can be regulated by the nervous system, it may already play a part in analyzing complex acoustic scenes. However, investigations of these questions have been hindered by a lack of experimental tools to assess the inner ear’s activity in relation to speech processing. Here, we develop a method to record otoacoustic emissions (OAEs) that relate to the harmonic structure of speech, a key feature of many speech parts. We use the novel tool to provide tentative evidence that the inner ear contributes to selective attention.

## Introduction

Humans have a remarkable ability to selectively listen to one of several competing speakers and understand them despite the interfering voices. The cognitive processes involved in these tasks are typically attributed to the auditory cortex that receives its inputs from lower-level neural processing centers. However, extensive feedback loops exist between the lower-level structures and the cortex. In particular, descending efferent nerve fibers transmit information from the auditory cortex to the superior olivary complex and to the cochlear nuclei, and through the olivocochlear bundle from the superior olivary complex to the inner ear (Pickles, 1988; Huffman and Henson, 1990; Winer et al., 1998).

The inner ear, or cochlea, detects sound vibrations by transducing them into electrical signals in the auditory nerve. However, the inner ear also aids the processing of sound. It spatially separates the different frequency components of a complex tone: high frequencies cause maximal vibration near the base of the organ and lower frequencies are detected at progressively more apical locations (Pickles, 1988; Robles and Ruggero, 2001; Reichenbach and Hudspeth, 2014). This spatial frequency decomposition is aided by an active process that mechanically amplifies weak signals, and thereby boosts the frequency selectivity. As a characteristic of the active process, the vibration amplitude at the peak location depends on the sound intensity in a compressively nonlinear manner.

The active process is mediated by the inner ear’s mechanosensitive outer hair cells. These cells are innervated by one of two types of the olivocochlear fibers, the medial ones. Activation of the medial olivocochlear (MOC) fibers can reduce the mechanical amplification provided by the outer hair cells (Guinan, 2006; Lopez-Poveda, 2018). Because each MOC fiber is tuned to a narrow frequency band, and because the innervation of the inner ear by these fibers displays a tonotopic arrangement, the reduction of cochlear amplification can potentially vary with frequency (Liberman and Brown, 1986; Brown, 1989; Lilaonitkul and Guinan, 2012). Computational models of the inner ear and efferent feedback have shown that the efferent feedback can contribute to speech processing through frequency-specific modulation of its mechanical activity (Messing et al., 2009; Clark et al., 2012). Experimental verification of such an effect remains, however, lacking.

The strength of the mechanical amplification in the cochlea, as well as its regulation through efferent feedback, can be assessed through distortion-product otoacoustic emissions (DPOAEs). A by-product of the active process’ compressive nonlinearity, DPOAEs are typically elicited by two pure tones of nearby frequencies *f*_1_ and *f*_2_, and emerge in particular at the cubic distortion product frequencies 2*f*_1_ – *f*_2_ and 2*f*_2_ – *f*_1_. By convention, the two primary frequencies are chosen such that *f*_1_ < *f*_2_. The cubic distortion product 2*f*_1_ – *f*_2_ is then below the two primary frequencies and is referred to as the lower-sideband distortion product. Conversely, the upper-sideband distortion product 2*f*_2_ – *f*_1_ is higher than *f*_1_ and *f*_2_.

Because of efferent feedback, DPOAEs from one ear are suppressed when stimulating the other ear with noise [MOC reflex (MOCR); Guinan, 1996, 2006]. However, an investigation of the role of efferent feedback for speech processing has been hampered by the complexity of speech. As opposed to the pure tones used for DPOAE measurements, speech is a broad-band, time-varying, and non-stationary signal, which obstructs an assessment of cochlear responses.

An important feature of speech is its harmonic structure. Many parts of speech are voiced, that is, they arise from vibration of the vocal folds. The vibration occurs at a fundamental frequency, typically between 100 and 300 Hz, and the resulting speech signal is dominated by that frequency as well as its higher harmonics. Neural activity in subcortical areas phase lock to this harmonic structure (Galbraith et al., 1995; Russo et al., 2004; Skoe and Kraus, 2010), and we have recently shown that these neural responses are modulated by selective attention to speech (Forte et al., 2017; Etard et al., 2019). Importantly, the attentional modulation of the brainstem response only emerged systematically when measuring it in response to running speech, while responses to short tones such as single vowels yielded inconclusive results (Galbraith et al., 1998; Choi et al., 2013; Lehmann and Schönwiesner, 2014).

Because of efferent feedback, the cochlear activity related to the harmonic structure of speech may already be modulated to aid its processing. In particular, the cochlear activity at locations that do not correspond to the harmonics of a speech signal might be reduced, which could help to reduce background noise. Computational modeling shows that such modulation of cochlear activity that depends on the cochlear location and therefore affects different frequency bands of the encountered sound differently can indeed aid with sound processing (Clark et al., 2012).

To investigate this issue, we develop a method to monitor the inner ear’s activity related to speech through DPOAEs that are matched to the harmonic structure (speech-DPOAEs). We then show that the speech-DPOAEs can be employed to investigate the role of the efferent feedback to the inner ear in selectively attending to one of two continuous speech signals, an ecologically highly relevant scenario.

## Materials and Methods

### Participants

A total of 24 healthy young volunteers (14 female, 10 male) aged between 18 and 26 years were recruited. All subjects were native English speakers and had no history of hearing or neurologic impairments. The experimental procedures were approved by the local Ethics Committee, and were performed in accordance with all relevant guidelines and regulations. Informed consent was obtained from all participants.

### Test environment

All testing was conducted in a sound-proof and semi-anechoic room. A personal computer (PC) controlled the audio presentation and data acquisition. Experiments were automated and instructions were presented to subjects through the PC; when prompted, subjects submitted responses using a keyboard. Sound stimuli were presented at a sampling frequency of 44.1 kHz through a high-performance sound card (RME Fireface 802) and delivered by an extended-bandwidth otoacoustic measurement system (ER10X, Etymotics) through probes placed in both ears of a subject. Each probe contains a microphone and three speakers which allow for a simultaneous presentation of the acoustic stimuli and the measurement of the acoustic emissions generated by the inner ear. OAEs were recorded through this system from the right ear, at the same sampling rate.

**Figure 1.**
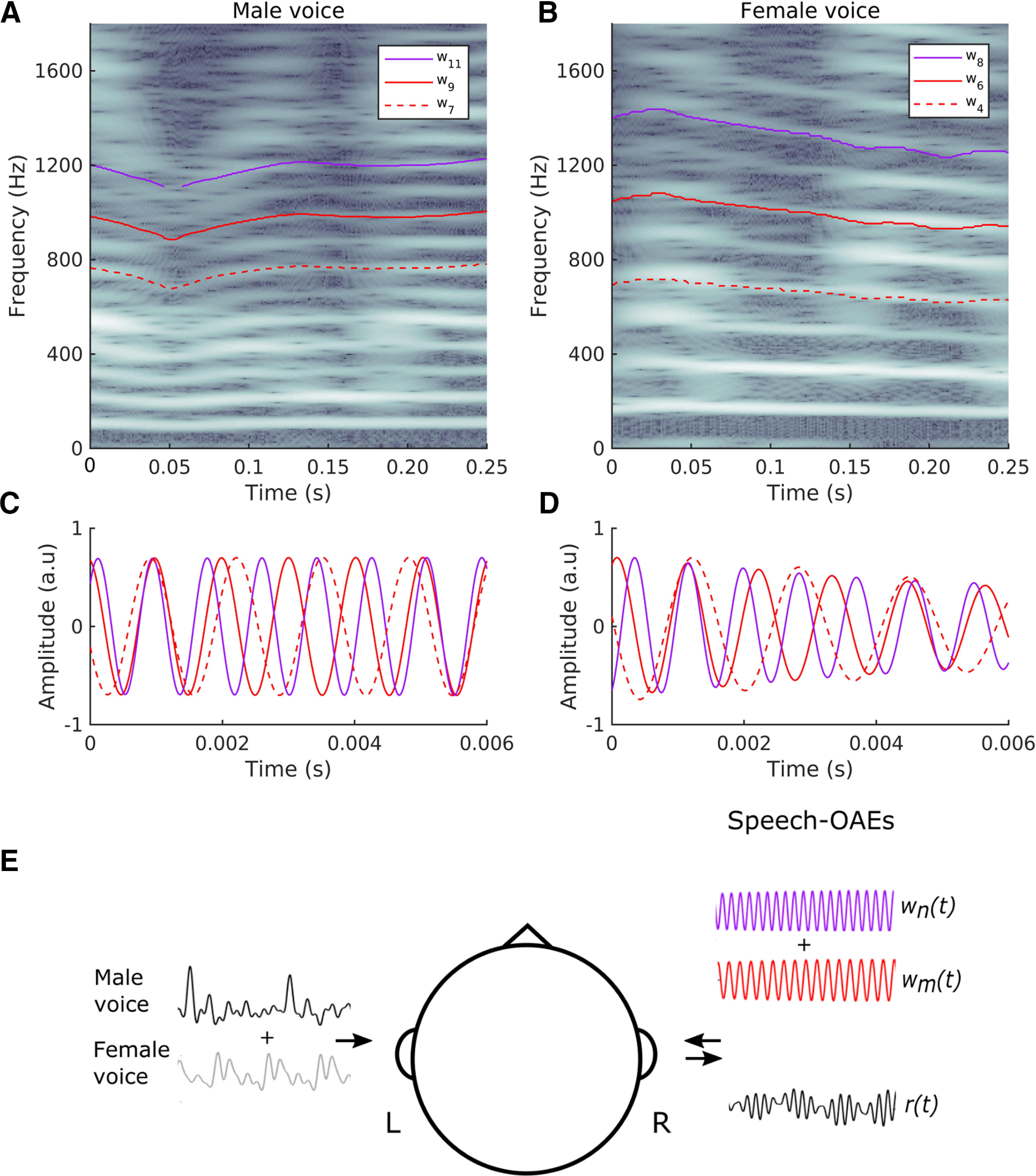
The waveforms used to elicit and detect speech-DPOAEs. ***A***, ***B***, The spectrogram of the voiced parts of a male speech signal (***A***) or a female speech signal (***B***) shows the harmonic structure, with a fundamental frequency and many higher harmonics (note that the colormap represents lower power as dark and higher power as white). ***C***, ***D***, The waveforms used to elicit and to detect the speech-DPOAEs to the male voice (***C***) and to the female voice (***D***). ***A***, ***C***, We measure speech-DPOAEs related to the male voice by constructing waveforms $w_{9}(t)$ (red line) and $w_{11}(t)$ (purple line) that oscillate at the 9th and 11th harmonics of the fundamental frequency of the speech signal, respectively. The lower-sideband speech-DPOAE then emerges at the 7th harmonic and is measured through cross-correlation with the corresponding waveform $w_{7}(t)$ (dashed red line). ***B***, ***D***, The speech-DPOAEs related to the female voice are elicited by waveforms $w_{6}(t)$ (red line) and $w_{8}(t)$ (purple line) that correspond to the 6th and 8th harmonics. The speech-DPOAE is found at the 4th harmonic, we measure it through the waveform $w_{4}(t)$ (dashed red line). ***E***, In our experiment, we presented subjects with speech stimuli to the left ear. The speech stimuli were either a single voice or two competing voices, a male and a female one. Two waveforms $w_{n}(t)$ and $w_{m}(t)$ that were derived from one of the speech stimuli were presented to the right ear. The microphone signal r(t) was recorded from the right ear as well, and the speech-DPOAE was derived from this recording.

Because OAEs are very faint signals and easily masked by other sounds, we presented the speech signals to the contralateral and not to the ipsilateral ear (Fig. 1*E*). We thereby tested whether attention to one of two speakers presented to the left ear would affect cochlear activity contralaterally.

Sound intensity was calibrated with an ear simulator (type 4157, Brüel & Kjær). The ER-10X instrument comes with a supply of single-use eartips that fit a variety of ear canal sizes. Precaution was taken to ensure the selection of an appropriate size for each participant and a correct fitting of the probe, as the eartip must seal the ear canal.

### Generation and measurement of speech-DPOAEs

We sought to measure DPOAEs related to the temporal fine structure of speech. We thereby used continuous natural speech that we obtained by recording a male and female speaker reading a story. The male voice had a fundamental frequency of 105 ± 6 Hz (mean ± SD), while the female voice had a fundamental frequency of 172 ± 10 Hz (mean ± SD). For a given voiced part of a speech signal, we therefore computed a fundamental waveform $w_{0}(t)$, that is, a temporal signal that varied at each time point *t* at the fundamental frequency $f_{0}(t)$ of the speech at that moment. The fundamental waveform was obtained from bandpass filtering the speech around the fundamental frequency. The latter was determined using the speech-analysis software package PRAAT (Boersma, 2002). The bandpass filter was a zero-phase sixth order IIR filter. The filter passbands were 0.5 SD above and below the mean fundamental frequency. For the non-voiced parts of a speech signal, the fundamental waveform was zero. We confirmed that the filter did not introduce any delays.

From the fundamental waveform we then obtained further waveforms whose instantaneous frequencies matched the higher harmonics of the fundamental frequency. In particular, we obtained the waveform $w_{n}(t)$ that corresponded to the *n*th harmonic of the fundamental frequency by applying a Hilbert-transform frequency shifter to the fundamental waveform $w_{0}(t)$ (Wardle, 1998). To this end, we first determined the analytical representation of the fundamental waveform $w_{0}(t)$, using the Hilbert transform $H\left[w_{0}(t)\right]$:

(1) $$W_{0}(t)=w_{0}(t)+i\cdot H\left[w_{0}(t)\right].$$

We then used the analytical representation to obtain a waveform whose instantaneous frequency was *n*-fold that of the fundamental waveform:

(2) $$w_{n}(t)=\left|W_{0}(t)\right|\text{cos}(arg\left[W_{0}(t)\right]*n).$$

We employed the waveforms that corresponded to the higher harmonics to elicit, as well as to measure, the speech-DPOAEs. We stimulated the ear of a subject with two waveforms $w_{m}(t)$ and $w_{n}(t)$ that tracked the *m*th and *n*th harmonics of the fundamental frequency, with *m* smaller but not much below *n* (Fig. 1*E*). The instantaneous frequencies of these two waveforms at time *t* were $mf_{0}(t)$ and $nf_{0}(t)$. They therefore elicited cubic distortion at the instantaneous frequencies $\left(2m-n)f_{0}(t\right)$ and $\left(2n-m)f_{0}(t\right)$ that corresponded to the frequency range of the waveforms $w_{2m-n}(t)$ and $w_{2n-m}(t)$.

This approach enabled us to assess cochlear activity at frequencies, and therefore at cochlear locations, that corresponded to the harmonic structure of speech. A traditional approach using constant primary frequencies *f*_1_ and *f*_2_, in contrast, would not have allowed to track cochlear activity at the temporally-varying harmonic structure.

However, regarding the analysis of the speech-DPOAE, the nonstationary nature of speech and the waveforms that we derived from it meant that we could not employ power spectra to identify the emissions. Instead, we employed a cross-correlation approach in which we compared the microphone recording with the expected cubic distortion waveforms that we computed from the speech signals as well, $w_{2m-n}(t)$ and $w_{2n-m}(t)$.

In our analysis, we focused on the lower sideband speech-DPOAE at the instantaneous frequency $\left(2m-n)f_{0}(t\right)$, as the lower sideband cubic DPOAE is the strongest in human ears (Probst et al., 1991). We measured this speech-DPOAE by cross-correlating the microphone recording r(t) (Fig. 1) with the waveform $\left(2m-n)f_{0}(t\right)$. Because the speech-DPOAE could have a phase shift with respect to the waveform $w_{2m-n}(t)$, we interpreted this cross-correlation as the real part of a complex cross-correlation C(τ) that depended on the delay τ. The imaginary part of this complex cross-correlation was computed as the cross-correlation of the microphone recording with the Hilbert transform of the waveform $w_{2m-n}(t)$. The complete complex cross-correlation thus followed as

(3) $$C(\tau )=N\overset{\infty }{\underset{-\infty }{\int }}r(t+\tau )\left\{w_{2m-n}(t)+iH\left[w_{2m-n}(t)\right]\right\}dt,$$

in which i denotes the imaginary unit and $H\left[w_{2m-n}(t)\right]$ is the Hilbert transform of $w_{2m-n}(t)$. The normalization coefficient *N* is determined such that the auto cross-correlations at zero lag equal 1. The delay of the speech-DPOAE could then be obtained from the delay at which the amplitude of C(τ) peaked, and the phase shift followed from the phase of C(τ) at that latency. We had previously developed a similar procedure to detect the brainstem response at the fundamental waveform of speech at a particular delay and phase shift (Forte et al., 2017).

DPOAEs are strongest for a ratio of the two primary frequencies of ∼1.2 (Abdala, 1996). They are easiest to measure for primary frequencies of ∼1 kHz or higher (Probst et al., 1991). We followed these recommendations and chose harmonics that were in this range. This additionally allowed us to make comparisons between pure-tone DPOAEs and speech-DPOAEs. For the male voice, we therefore employed the 9th and 11th harmonics, that is, the waveforms $w_{9}(t)$ and $w_{11}(t)$ (Fig. 1*A*,*C*). The lower-sideband speech-DPOAE emerged accordingly in correlation to the waveform $w_{7}(t)$, and had a frequency of 735 ± 26 Hz (mean ± SD). For the female voice, we used the 6th and 8th harmonics, that is, the waveforms $w_{6}(t)$ and $w_{8}(t)$ (Fig. 1*B*,*D*). The lower-sideband speech-DPOAE was then at the 4th harmonic ($w_{4}(t)$), with a frequency of 692 ± 31 Hz (mean ± SD).

The different harmonics of the fundamental frequency that we employed for the male and for the female speech fell into two classes, resolved and unresolved. Because of the cochlea’s logarithmic mapping between spatial location and best frequency, only the lower harmonics are resolved. The upper limit for resolved harmonics is considered to be the 9th (Micheyl and Oxenham, 2007). The harmonics of the fundamental frequency of the female speaker were therefore more resolved than those of the male speaker.

### Experimental design

We first measured pure-tone DPOAEs from the subject’s right ear. We therefore employed primary frequencies of $f_{1}=1$ kHz and $f_{2}$ = 1.2 kHz that were presented at 60-dB SPL for a duration of 30 s.

We then measured speech-DPOAEs while subjects listened to speech both in isolation and in noise. To this end, we employed different speech segments, all of which lasted 2 min. Some speech segments consisted only of the male or of the female voice, while others had both voices mixed together. The speech-DPOAEs were always measured from the right ear. To increase the signal-to-noise ratio of the recorded OAEs, the speech stimuli where not applied to the right ear as well, but exclusively to the left ear. Because the MOCR pathway is crossed, stimulation of a given ear can also modulate the contralateral inner ear activity. Selective attention to one of two speakers heard in a given ear may similarly modulate the activity of the contralateral cochlea, and thus modulate the speech-DPOAEs recorded from that ear. The speech segments were presented at 60-dB SPL, and the waveforms to elicit the speech-DPOAEs at a somewhat lower intensity of 57-dB SPL. The intensities were chosen to avoid evoking the middle-ear muscle reflex, as well as to enable subjects to focus on the speech signals without being distracted by the speech-DPOAE measurement.

We first familiarized the subject with the speech-DPOAE measurement and with the attention task. To this end subjects were presented with a speech stimulus that contained both the male and the female voice. Speech-DPOAEs related to the male speech were simultaneously elicited in the contralateral ear. The subject was instructed to listen to the male speaker and to ignore the female speaker. To verify attention, the subject then answered three comprehension questions regarding the male speech. This was then repeated, but subjects were asked to attend the female speech, while speech-DPOAEs related to the female voice were elicited.

We then measured speech-DPOAEs to speech in isolation. To this end, we employed one speech stimulus that consisted of the male voice, as well as another stimulus that contained the female voice. Each stimulus was followed by three comprehension questions.

The potential influence of selective attention to speech on the speech-DPOAEs was then assessed. We employed 12 segments with competing speech, that is, segments that contained both the male and the female voice. During each segment the subject was asked to attend either the female or the male speaker. Speech-DPOAEs related to either the male or the female speaker were measured from the contralateral ear. The segment was then presented again. The same speech-DPOAEs were measured, but the subject was asked to attend the other speaker. The attended and ignored segments were therefore paired. In this way we obtained three recordings of speech-DPOAEs to the male voice, both when that voice was attended and when it was ignored. Analogously we measured speech-DPOAEs to the female voice for three speech segments, both when the female voice was attended and when it was ignored. After each speech segment the subject answered three comprehension questions. The order of the attentional focus, as well as the order in which speech-DPOAEs to the male and the female voice were measured, was determined randomly per subject.

### Preprocessing and analysis of OAEs

The pure-tone DPOAEs were first analyzed using a power spectrum of the microphone recording. The noise floor of the recording was computed from the spectral amplitudes within 30–70 Hz to each side from the DPOAEs. A pure-tone distortion product was considered to be significant if the DPOAE amplitude was larger than the 95th percentile of the noise.

Second, the pure-tone DPOAEs were processed in a manner that was comparable to the speech-DPOAEs. For this purpose, a sinusoidal waveform at the frequency of the lower sideband distortion product of 800 Hz was created. This waveform was then cross-correlated with the microphone recording following Equation 1. The envelope of the complex cross-correlation was smoothed with a moving-average filter of 199 samples. The noise level was computed by following the same procedure but using the unrelated nearby frequency of 900 Hz. The pure-tone DPOAEs was considered to be significant if the peak correlation amplitude in the range 0–7 ms was larger than the 95th percentile of the noise.

For computing speech-DPOAEs, the first and last three seconds of each recording were removed to eliminate transient activity. The envelope of the complex cross-correlation (1) was smoothed with a moving-average filter of 199 samples. To determine the equipment delay, the recording was also cross-correlated with the eliciting harmonics; the delay of the maximum correlation corresponded to the equipment delay. The recordings were compensated for this equipment delay.

The noise level of the speech-DPOAEs was determined as the 95th percentile of the amplitudes of the complex cross-correlation (1) in the temporal regions of −750 to −70 and 70 to 750 ms. A speech-DPOAE was considered to be significant if the peak amplitude of the complex cross-correlation (1) in the range of delays between 0 and 7 ms was larger than the noise level.

### Comprehension scores

Speech comprehension was assessed through multiple-choice questions. The questions came in two formats: 60% of them had four possible answers, and the remaining 40% of questions had two possible answers. The comprehension score of a subject was computed as the proportion of correct answers to the questions posed during the selective attention task. Two participants did not score above the chance level.

### Attentional modulation of the speech-DPOAEs

We analyzed the effect of selective attention on both the amplitude and the latency of the speech-DPOAEs. We performed this analysis for each of the three segments of pairs for which we recorded speech-DPOAEs related to the male voice, as well as for each of the three segments of pairs for which speech-DPOAEs related to the female voice were measured.

Denote by $r_{M}^{\left(A\right)}$ the peak amplitude of the speech-DPOAE related to the male voice when that voice was attended, and by $r_{M}^{\left(I\right)}$ the peak amplitude of the speech-DPOAE related to male voice when that voice was ignored. We then defined the relative attentional modulation of the speech-DPOAE related to the male voice as the difference between the two amplitudes, divided by the average amplitude: $A_{M}=2\frac{r_{M}^{\left(A\right)}-r_{M}^{\left(I\right)}}{r_{M}^{\left(A\right)}+r_{M}^{\left(I\right)}}$. A positive relative attentional modulation signified a larger speech-DPOAE related to the male voice when it was attended, and a negative value implied a larger response when the male voice was ignored. Analogously, we defined the peak amplitudes $r_{F}^{\left(A\right)}$ and $r_{F}^{\left(I\right)}$ of the speech-DPOAE related to the female voice when this voice was attended respectively ignored. These coefficients yielded the relative attentional modulation of the speech-DPOAE related to the female voice, $A_{F}=2\frac{r_{F}^{\left(A\right)}-r_{F}^{\left(I\right)}}{r_{F}^{\left(A\right)}+r_{F}^{\left(I\right)}}$.

The relative attentional modulation of the speech-DPOAE related to the male voice was computed for all three corresponding recordings separately, that is, for the three pairs of recordings in which the participants once attended the corresponding speaker and once ignored them. We then averaged the obtained coefficients to obtain a single value per subject. The same procedure was employed for the relative attentional modulation of the speech-DPOAE related to the female voice.

To test the attentional modulation at the level of individual subjects, we split each of the recording segments into 10 consecutive intervals and computed the corresponding speech-DPOAEs. For each interval, we determined the amplitude of the speech-DPOAE at the latency of the peak amplitude of the corresponding segment. For each pair of the intervals, with one interval corresponding to the task of attending the male voice and the other interval related to the task of attending the female voice, we then computed the relative attention modulation coefficient, either *A_F_* or *A_M_*, depending on which speech-DPOAE was measured. We then performed one-sided *t* tests on all the obtained modulation coefficients *A_F_* from a given subject to determine whether the relative attentional modulation with respect to the female voice was significantly above zero. Analogously, we conducted one-sided *t* tests on the coefficients *A_M_* to establish their statistical significance at the level of individual subjects.

To assess the potential modulation of the latency through selective attention, we computed the difference in the latency of the speech-DPOAE when the corresponding speech was attended and when it was ignored. The difference was computed for each of the three corresponding stimuli separately, and the average of the differences was taken subsequently.

### Exclusion of subjects and statistical analysis

We performed statistical tests both regarding amplitude and latencies of the speech-DPOAEs when speech was presented in isolation, as well as regarding the amplitude and latencies of the speech-DPOAEs when participants attended one of two competing speakers.

To assess the attentional modulation, we excluded data from two subjects who had non-significant pure-tone DPOAEs and from another two subjects whose answers to the speech comprehension questions did not exceed chance level.

Regarding the attentional modulation of the speech-DPOAEs related to the male voice, we excluded the data from four further subjects whose corresponding speech-DPOAEs were non-significant. With respect to the attentional modulation of speech-DPOAEs related to the female voice, data from three subjects whose corresponding speech-DPOAEs did not reach significance were excluded.

The amplitudes and latencies of the different speech-DPOAEs, as well as their changes because of switching attention, were then checked for normality through the Kolmogorov–Smirnov test. Non-parametric tests were used for the hypothesis testing as the data were not normally distributed and as the sample size was small. We further used a bootstrap analysis to estimate the sampling distributions of the mean attentional effects, to compute the confidence intervals for estimation inference, and to test the stability of the results. In particular, we used a random sampling with replacement procedure for 10,000 resamples and performed bootstrap hypothesis tests for the mean. We used a significance level of 0.05.

### Data availability

**Figure 2.**
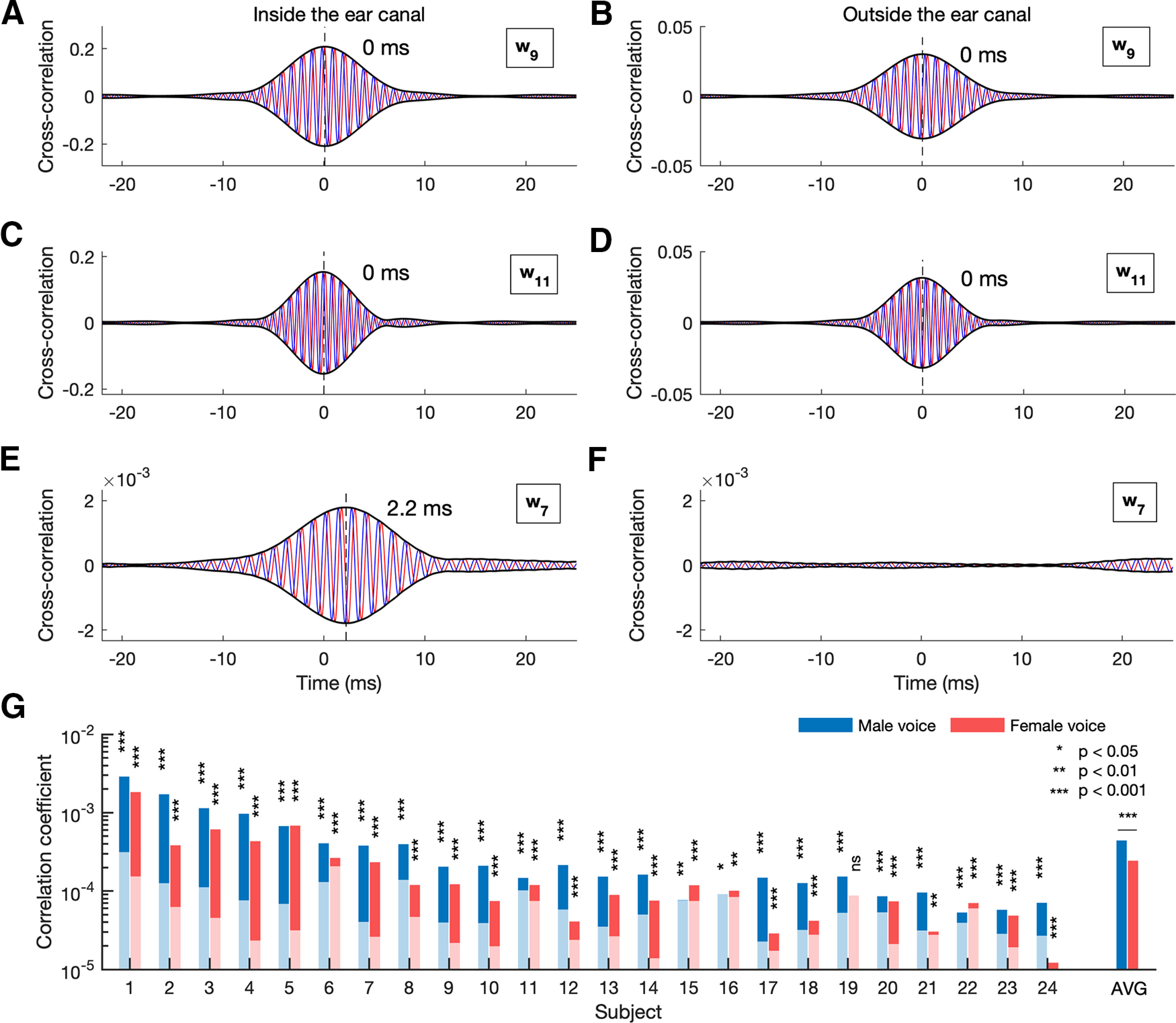
Measurement of speech-DPOAEs. ***A–D***, Complex cross-correlations of the microphone recording of a representative subject with the stimulating waveforms for the male voice [$w_{9}\left(t\right)$ and $w_{11}(t$)], when the probe is placed inside the ear canal (***A***, ***C***, respectively) and when it is hold outside the ear (***B***, ***D***, respectively). The data from the representative subject show that the complex cross-correlation of each stimulation waveform with the microphone recording peaks at 0 ms (blue: real part, red: imaginary part, black: amplitude). These peaks occur both when the probe is placed inside (***A***, ***C***) as well as outside the ear canal (***B***, ***D***). ***E***, An OAE is measured by computing the complex cross-correlation between the microphone recording and the waveform $w_{7}(t)$ that corresponds to the lower-sideband distortion. We refer to this emission as a speech-DPOAE. The amplitude peaks at a latency of 2.2 ms (dashed line). ***F***, The speech-DPOAE measured outside the ear canal. When the probe is placed outside the ear canal, the cross-correlation does not show a significant peak, demonstrating that no emission could be detected. ***G***, Individual peak values of speech-DPOAEs for male and female speech in isolation. In most subjects the amplitude of the speech-DPOAE (darker bar) was significantly above the noise floor (lighter superimposed bar). The population average of the speech-DPOAE related to the male voice was significantly larger than that related to the female voice.

The speech stimuli, the corresponding harmonic waveforms, as well as the recordings of the pure-tone and speech-DPOAE from all participants are available on figshare (https://doi.org/10.6084/m9.figshare.12738515). The repository also contains an example script for computing the speech-DPOAE as presented in Figure 2*E*.

## Results

We sought to measure OAEs that were related to the harmonic structure of continuous non-repetitive speech, an ecologically relevant stimulus. We therefore devised a method to measure such speech-DPOAEs by eliciting distortion products from waveforms that tracked particular harmonics of the fundamental frequency of the speech signal (Fig. 1). The distortion emerged then at nearby harmonics.

We first verified the presence of a particular stimulation waveform in the microphone recording by cross-correlating the recording with that waveform. We found that we could thereby indeed measure the two waveforms that were used to stimulate the OAEs: each of them caused a peak in the corresponding cross-correlation, at a delay of 0 ms (Fig. 2*A–D*). These peaks emerged whether or not the probe for stimulating the ear and measuring the sound pressure was placed inside or outside the ear canal, since these signals were produced by the probe itself.

The speech-DPOAE was then measured analogously by cross-correlating the obtained microphone recording with the waveform of the harmonics that corresponded to a distortion product. The speech-DPOAE emerged then as a peak in that cross-correlation (Fig. 2*E*). As a control, this peak disappeared when the probe used to measure the speech-DPOAEs was placed near but outside the ear canal (Fig. 2*F*).

When subjects listened to a single speaker, we found that we could record significant speech-DPOAEs in all 24 subjects: all recordings except for one that was related to the female voice were significant (Fig. 2*G*). The amplitude of the speech-DPOAEs was 3.4e-4 ± 8e-5 (population average over male and female voices and standard error of the mean). The speech-DPOAE related to the male voice, was, however, significantly larger than that related to the female voice (*p *=* *0.0002; two-tailed two-sample Wilcoxon signed-rank test; Fig. 2*G*). It also had a larger variance (*p *=* *0.02; Bartlett’s test). The latency of the speech-DPOAEs was 2.3 ± 0.2 ms (population average over male and female voices and standard error of the mean). It did not differ between the male and the female voice (*p *=* *0.8; two-tailed two-sample Wilcoxon signed-rank test).

**Figure 3.**
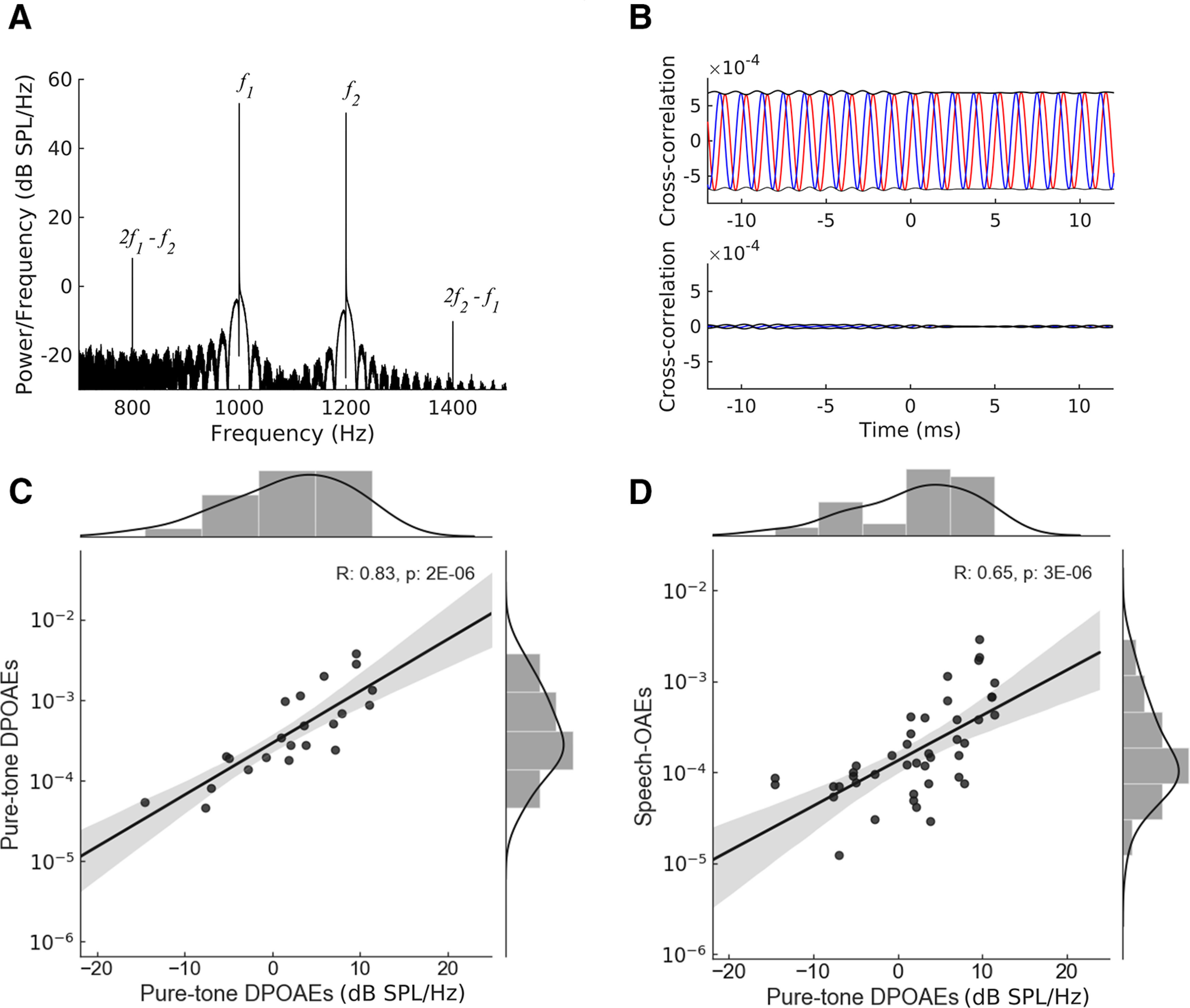
Relation of speech-DPOAEs to pure-tone DPOAEs. ***A***, The power spectrum of the microphone recording in response to pure tones of a representative subject. Pure-tone DPOAEs were measured in response to the two primary frequencies $f_{1}=1$ kHz and $f_{2}$ = 1.2 kHz, and emerged at the cubic distortion frequencies $2f_{1}-f_{2}$ and $2f_{2}-f_{1}$. ***B***, The cross-correlation of the lower-sideband $2f_{1}-f_{2}$ with the microphone recording of the same subject shows an amplitude of about 5e-4 (upper panel), significantly higher than that obtained when the probe is placed outside the ear canal (lower panel). ***C***, Comparison between the lower-sideband pure-tone DPOAEs analyzed through the two methods presented in ***A***, ***B***. The amplitude of the pure-tone DPOAEs when analyzed through the cross-correlation method (ordinates), strongly correlated with the amplitude obtained from the power spectrum across subjects (abscissas). ***D***, Comparison between the pure-tone DPOAEs obtained through the power spectrum and the speech-DPOAEs peak responses. The amplitude of the speech-DPOAEs was strongly correlated, across subjects, to the amplitude of the lower-sideband DPOAE $2f_{1}-f_{2}$ as well.

To compare the speech-DPOAEs to conventional OAEs, we also measured pure-tone DPOAEs (Fig. 3). We first analyzed the recordings through computing the power spectrum, which showed peaks at the DPOAE frequencies (Fig. 3*A*). Using this type of analysis, we found that the upper sideband distortion product $2f_{2}-f_{1}$ was measurable in 14 of the 24 subjects, while the lower-sideband distortion product $2f_{1}-f_{2}$ could be detected in all subjects but two. The power spectrum of the upper sideband distortion product $2f_{2}-f_{1}$ was 2 ± 1-dB SPL/Hz (population average and standard error of the mean), and that of the lower-sideband distortion product $2f_{1}-f_{2}$ reached −11 ± 1-dB SPL/Hz (population average and standard error of the mean).

To relate the DPOAE measurement to the speech-DPOAEs, we also analyzed the lower-sideband distortion product $2f_{1}-f_{2}$ using the cross-correlation method (Fig. 3*B*). We found that the cross-correlation of sinusoidal oscillations at the distortion frequency $2f_{1}-f_{2}$ yielded significant results in all but one subject. Moreover, the amplitude of the cross-correlation for this DPOAE was strongly related to its power spectrum across the different subjects (Pearson correlation coefficient *r *=* *0.83, *p *=* *2e-6; Fig. 3*C*). This strong correlation showed that a DPOAE power spectrum of 10 dB/Hz, for instance, corresponded to a cross-correlation amplitude of approximately 1e-3.

We further investigated the relation between the power spectrum of the distortion product $2f_{1}-f_{2}$ with the amplitude of the speech-DPOAEs across the different participants (Fig. 3*D*). We found that these two measures exhibited a strong and significant correlation as well (Pearson correlation coefficient *r *=* *0.65, *p *=* *3e-6). As an example, a DPOAE power spectrum of 7-dB SPL/Hz corresponded on average to a speech-DPOAE of an amplitude of 1e-3.

Armed with the ability to monitor cochlear activity related to the harmonic structure of speech through the speech-DPOAEs, we then sought to employ them to investigate speech processing in the cochlea. We focused on an important aspect of speech-in-noise comprehension, namely selective attention to the target voice.

To this end, we presented subjects with both a male and a female voice in one ear, while measuring speech-DPOAEs from the contralateral ear. Subjects were instructed to sometimes attend the male and sometimes the female voice, and were asked comprehension questions regarding the target speech signal. The participants achieved a comprehension score of 80 ± 13% (population mean ± SD), demonstrating that they were able to maintain a high level of attention.

**Figure 4.**
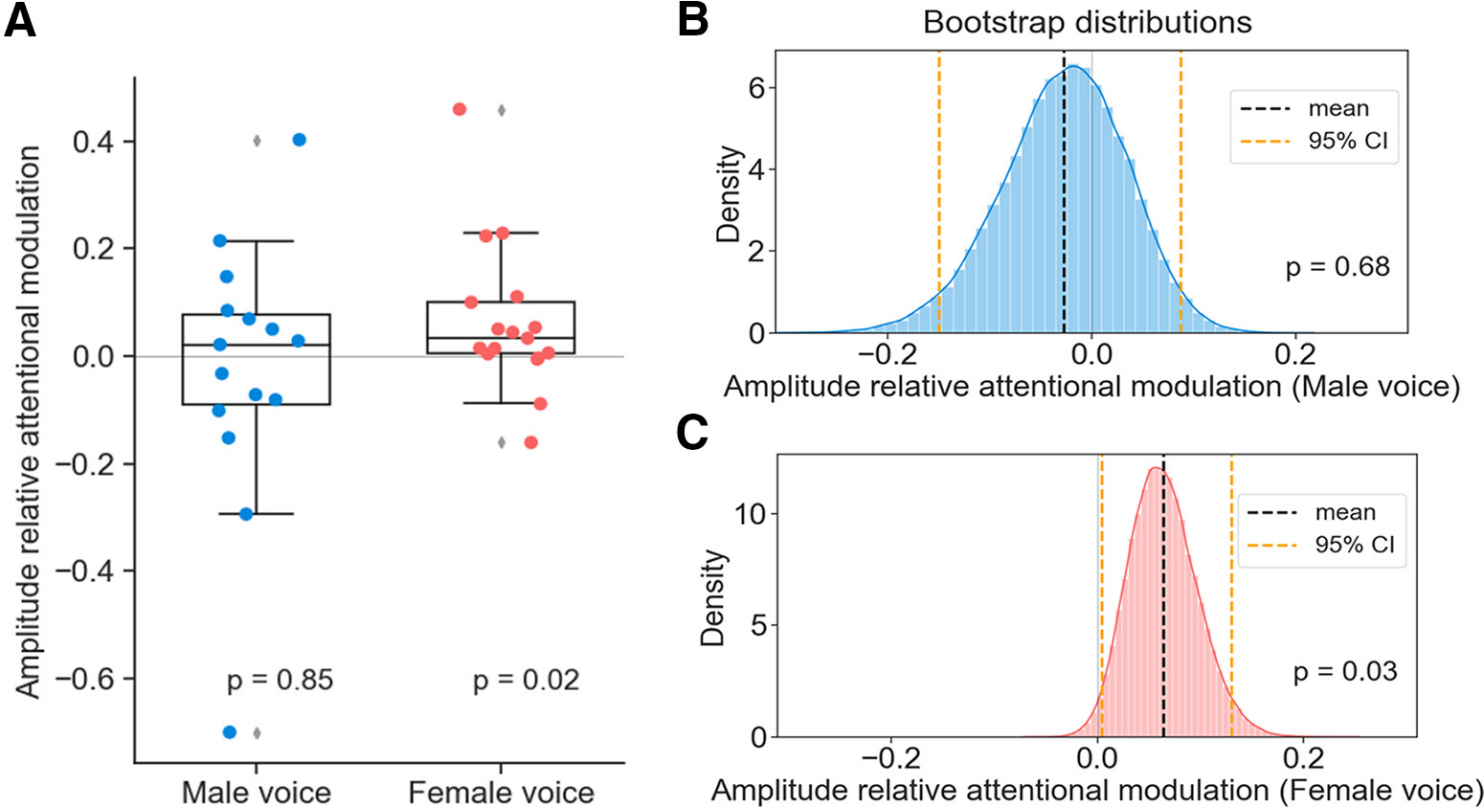
Attentional modulation of speech-DPOAEs. Individual attentional modulations of speech-DPOAEs to male and female voices (***A***; the diamond markers represent outliers) and bootstrap distributions of the mean amplitude relative attentional modulation to the male voice (***B***) and female voice (***C***). ***A***, The relative attentional modulation of the speech-DPOAEs related to the male voice is not significantly different from zero. Speech-DPOAEs related to the female voice are, however, significantly larger when the female voice is attended than when it is ignored. ***B***, ***C***, The bootstrapping procedure confirms that the results are stable, and that the attentional modulation related to the female voice has a large intersubject variability (***C***).

We then analyzed the magnitude of the speech-DPOAEs for each voice and how it was modulated by selective attention. We found that the speech-DPOAEs related to the female voice were larger when the subject attended the female speaker than when that voice was ignored (Fig. 4*A*). The relative attentional modulation of the speech-DPOAE related to the female voice, $A_{F}$, was 0.064 and was significantly greater than zero (*p *=* *0.02, two-tailed one-sample Wilcoxon signed-rank test). The statistical significance remained when removing two outliers (*p *=* *0.01, two-tailed one-sample Wilcoxon signed-rank test).

To test the stability of the results and to derive an additional estimate of the mean relative attentional modulation, we performed a bootstrapping procedure (Fig. 4*C*). The 95% confidence interval for the mean attentional modulation ranged from 0.004 to 0.13. The bootstrapped one-sided *p* value of the estimated population mean was 0.03.

However, regarding the speech-DPOAEs related to the male voice, the relative attentional modulation $A_{M}$ was not significantly different from zero (*p *=* *0.8, two-tailed one-sample Wilcoxon signed-rank test). The removal of the outliers rendered a similar *p* value (*p *=* *0.7, two-tailed one-sample Wilcoxon signed-rank test). These results were confirmed by the bootstrapping, that yielded 95% confidence intervals for the mean modulation of [−0.15, 0.087], and no significant difference from 0 (*p *=* *0.65; Fig. 4*B*).

At the level of individual subjects, two subjects showed significant relative attentional modulations to the male voice (*A_M_*), with *p* values of 2e-5 and 3e-4. One further subject showed significant relative attentional modulation to the female voice (*A_F_*), with a *p* value of 0.02. The remainder of the attentional modulation coefficients were statistically insignificant, with *p* values between 0.06 and 0.9.

## Discussion

We developed a method to measure speech-DPOAEs, namely OAEs that were related to the harmonic structure of a speech signal. These OAEs were elicited by waveforms whose instantaneous frequency corresponded to that of particular harmonics of the fundamental frequency of the voiced parts of speech. They elicited distortion at other harmonics, and we measured these distortions by cross-correlating the microphone recording with the corresponding waveforms.

We compared the speech-DPOAEs to conventional pure-tone DPOAEs, and found that, across the different subjects, the amplitudes of the speech-DPOAEs were correlated to those of the pure-tone DPOAEs. Moreover, analyzing pure-tone DPOAEs in a manner that was comparable to the speech-DPOAEs showed that the amplitude of the speech-DPOAEs was comparable to that of the pure-tone DPOAEs. This suggests that the speech-DPOAEs and pure-tone DPOAEs have indeed a common origin in the cochlea.

Because the fundamental frequency of speech varies over time, the harmonics vary as well. The stimuli that we employed to elicit speech-DPOAEs, as well as the speech-DPOAEs themselves, were therefore not pure tones, but had a broader frequency spectrum. This enabled us to obtain the latency of the speech-DPOAEs, which provides further information on the origin of the emissions.

OAEs have been found to consist of two components, one with a long latency of many cycles and another with a short one of maximally a few cycles of delay (Knight and Kemp, 2001; Robles and Ruggero, 2001; Bergevin et al., 2008). The two different components may arise through different mechanisms of how the backward-traveling wave in the cochlea is generated (Shera and Zweig, 1991; Talmadge et al., 1999; Kalluri and Shera, 2001), or through different propagation mechanisms of the OAEs in the cochlea (Ren, 2004; He et al., 2007, 2008; Lutman et al., 2008; Reichenbach et al., 2012). The lower-sideband of pure-tone DPOAEs is dominated by the long-latency component, while the upper-sideband consists mainly of the short-latency component.

We found a latency of the speech-DPOAEs of only 2.3 ms, corresponding to ∼1.6 cycles. Although the speech-DPOAEs result from the lower-sideband distortion, their short delay reveals that they are dominated by the short-latency component. This deviation from the behavior of pure-tone DPOAEs may reflect the varying frequency of the speech-DPOAEs, which can introduce negative interference in the long-latency component. Because the phase of the latter changes rapidly with frequency, variation in frequency can indeed lead to significant cancellation effects. The phase of the short-latency component, in contrast, depends barely on frequency, except for the phase changes associated with the varying primary frequencies themselves (Reichenbach and Hudspeth, 2014). The fundamental frequency of speech does, however, not vary greatly, largely eliminating negative interference.

The speech-DPOAE related to the male voice had a larger amplitude than that related to the female voice. This behavior might reflect the different frequency ratios between the waveforms that we used to elicit the different speech-DPOAEs. Pure-tone DPOAEs have been found to be largest when the ratio of the primary frequencies $f_{1}$ and $f_{2}$ is $f_{2}/f_{1}\approx 1.2$ (Probst et al., 1991). The harmonics that we used to elicit the speech-DPOAEs related to the male voice, had a frequency ratio of 1.2, while the frequency ratio for the waveforms that yielded the speech-DPOAEs related to the female voice was slightly higher, 1.33. The higher ratio likely led to a smaller amplitude of the speech-DPOAEs that were related to the female voice.

To investigate whether the speech-DPOAEs that we measured could in fact be used to investigate the effects of speech processing on the cochlea, we employed the speech-DPOAEs to study whether they were affected by selective attention to one of two competing voices. We found that the speech-DPOAEs related to the female voice were larger when the female voice was attended than when it was ignored. The speech-DPOAEs related to the male voice were not affected by attention.

We can speculate that the lack of a significant effect for the male voice might reflect the poorer resolvability of the target harmonics. The speech-DPOAEs that were related to the female voice tracked the resolved harmonics. Their measurement therefore allowed to test whether cochlear activity at the locations of the resolved harmonics was modulated by selective attention. In contrast, the speech-DPOAEs for the male voice were related to unresolved harmonics. Our observation of an attentional modulation of the speech-DPOAEs related to the female, but not of those related to the male voice, is therefore consistent with an attentional modulation of cochlear activity regarding the resolved but not the unresolved harmonics of speech. In particular, the cochlea appears to facilitate selective attention to a voice through a larger mechanical response at the locations of the resolved harmonics, but not at the unresolved harmonics. Because only the resolved harmonics can be differentiated in the cochlea, an attentional modulation that aims to reduce background noise can indeed only sensibly operate on the resolved and not on the unresolved harmonics.

The attentional effect on the amplitude of the speech-DPOAE related to the female voice that we observed is only 6.4% on average, corresponding to 0.54 dB. Although this effect is small, it is comparable to the MOC reflex that is elicited by broadband noise and changes DPOAE magnitudes between 0.5 and 2 dB (Chéry-Croze et al., 1993; Timpe-Syverson and Decker, 1999; Sun, 2008). Intermodal attention such as between attending to an acoustic and to a visual signal has been found to have similar or smaller effects on the DPOAE amplitude (Wittekindt et al., 2014; Beim et al., 2018).

The bootstrap analysis of the data validated the stability of the attentional modulation effect. Nevertheless, the large confidence interval for the mean attention estimate suggests considerable uncertainty as well as a large interindividual variability, in lines with recent observations on selective attention modulation of cochlear function when attending to tones (Beim et al., 2018).

The attentional modulation of cochlear activity related to the harmonic structure of speech is reminiscent of the attentional effect on the brainstem response to voiced speech. As we have shown recently, the brainstem response at the fundamental frequency of continuous speech is modulated by selective attention (Forte et al., 2017; Etard et al., 2019; Saiz-Alía et al., 2019). In particular, the response is larger when the voice is attended than when it is ignored. Because of the nonlinearities in the inner ear and in the neural processing, the brainstem response at the fundamental frequency reflects cochlear activity at higher harmonics (Saiz-Alía and Reichenbach, 2020). The attentional modulation of the cochlear activity for which we have provided evidence here may contribute to the attention effect seen in the brainstem response.

DPOAEs and other types of OAEs have been employed previously to investigate how cochlear activity can be affected by selective attention, such as auditory versus visual attention, but have yielded inconclusive results that include both positive (Giard et al., 1994; Maison et al., 2001; de Boer and Thornton, 2007; Walsh et al., 2008; Harkrider and Bowers, 2009; Smith et al., 2012; Srinivasan et al., 2014; Wittekindt et al., 2014) and negative findings (Avan and Bonfils, 1992; Beim et al., 2018, 2019). Potential confounds in these measurements were a task-irrelevance of some of the stimuli that were used for eliciting the OAEs and a difficulty to assign attention tasks in different modalities that were balanced in perceptual load and working memory.

Our method of assessing the attentional modulation of the inner ear’s activity relates directly to natural speech processing. The task of selective attention to speech was naturalistic with a high ecological validity, and with a high perceptual load. This factor may have contributed to our positive finding regarding a modulation of speech-DPOAEs through selective attention. However, because of the small sample size, the lack of effect for the male condition and the large margin of error of the effect, the evidence for attentional modulation that we obtained still needs to be treated with caution. Moreover, the modulation by selective attention was, at the level of individual subjects, only significant in a few cases. While we believe that the speech-DPOAEs that we introduced open a promising path to study selective attention to speech in the cochlea, further studies are required to replicate our findings, to firmly establish the attentional modulation, to investigate the impact of the ratio of the primary frequencies on the amplitude of the resulting speech-DPOAEs, and to investigate to which degree resolved versus unresolved harmonics lead to differences in the attentional modulation.

In conclusion, the speech-DPOAEs that we have developed here provide a novel tool to measure inner-ear activity related to the processing of naturalistic speech. In particular, this enables to assess aspects of speech processing such as selective attention in a manner that fosters sustained attention of a participant and avoids potential neural adaptation to repeated stimuli. We therefore expect speech-DPOAEs and related complex OAEs to become a useful tool in further exploring how the inner ear contributes to the processing of complex real-world acoustic signals. They may also be relevant for a better understanding and diagnosis of poorly understood hearing impairments such as cochlear neuropathy or speech-in-noise deficits.

## Synthesis

Reviewing Editor: Christine Portfors, Washington State University Decisions are customarily a result of the Reviewing Editor and the peer reviewers coming together and discussing their recommendations until a consensus is reached. When revisions are invited, a fact-based synthesis statement explaining their decision and outlining what is needed to prepare a revision will be listed below. The following reviewer(s) agreed to reveal their identity: Alain de Cheveigné.

The reviewers agree that the manuscript presents a novel method of recording cochlear distortion that would be of interest to the community if there was further clarification of details in a revised manuscript. The manuscript tests an interesting hypothesis; that selective attention-dependent processing extends to the cochlea. However, both reviewers brought up significant concerns with the sample size, certain aspects of the results, and interpretation of the data that need to be addressed. In particular, the reviewers were concerned with fragility of the experiments thus rigor and reproducibility should be addressed. It was also suggested that one route in revising the manuscript may be to re-focus it on the novel technique and have less emphasis on testing the specific hypothesis.

Reviewer 1:

The paper is written with obvious care, it targets a very interesting hypothesis (that selective attention-dependent processing extends to the cochlea), and the experiments are ingenious and for most part seem well-executed. However, some aspects need clarifying (see details below).

Isolating the phenomenon of interest, attention-dependent distortion product otoacoustic emissions (DPOAEs) produced by interactions between partials of natural speech in the presence of a second competing voice, is technically challenging. However, the nature of the challenges may not be immediately obvious to the reader: the paper would be improved by adding a discussion of the options and constraints. For example, the reader might wonder why you don’t just measure DPOAEs from speech. Or, having understood why it’s better to measure them from 2 partials, they might wonder why put speech in the other ear. Answers may be pretty obvious, but the reader will appreciate some gentle guidance.

A critical assumption is that the level of DPOAEs between pairs of tones in one ear depends on attentional state to stimuli in the other ear. It would be nice to know more. Do DPOAEs even depend on whether or not there is contralateral stimulation with speech? On whether the stimulation is single-voice or double voice? If single-voice, on whether the primaries match that voice? It would be nice to have answers if any of your recordings allow them. Negative answers to any of these questions would call into question your main results. Conversely, asking them, and getting positive answers, would make your story more convincing.

Key methodological details are missing, in particular the critical step by which harmonic waveforms w_n(t) are derived from the filtered fundamental. The reference cited (Wardle 1998) mentions ‘frequency shifting’ but this is not what is needed here. Rather, you need scaling by a factor n. I have no idea how you do that. Also, I’m unsure as whether the phase of w_n(t) is important (for estimation of phase of the complex cross-correlation function), and if so, in what way the procedure ensures a proper value.

Details:

l19: ‘speech-DPOAE’ might be better than ‘speech-OAE’

l65: ‘cubic distortions’ --> ‘cubic distortion frequencies’. Readers familiar with distortion products (probably not that many) might be comfortable with the formulas, but for others it might make things easier to write ‘f1 - (f2-f1)’ and ‘f2 + (f2-f1)’ and paraphrase in English (e.g. ‘cubic distortion products consist in a component at (f2-f1) below the lower primary and a component at (f2-f1) above the higher primary’).

l85: not sure what is meant by ‘harmonic structure is modulated’

l99 and following: you first say that sound stimuli are presented to both ears, and then that speech is presented to the left ear. This is confusing because you haven’t introduced the DPOAE-inducing stimulus. Either do so earlier, or delay mentioning the speech signal to later.

l104: give more information about the acoustics. How are the probes constructed, were they sealed in the ear canal, etc.

l106: describe the speech stimulus in one place (i.e. move stuff from l140 etc. here).

Fig. 1: A, B: a bit confusing because it is common for high power to be represented as black rather than white. Harmonics appear aliased and don’t quite fit the colored lines. Panels C, D are very confusing (Are these cross-correlation functions? If so, the abscissa should be lag. What are we supposed to see?). Caption: describe each panel before telling us what is worth noting.

l111: this raises several potential issues. One is how does the filter track the time varying F0? Another is the latency of the filter (or at least its smoothing properties): might these induce a mismatch? A third is the fact that the filter is (presumably) time varying. A filter is normally assumed to be time-invariant, things are less straightforward if it varies. Perhaps add a sentence or two to reassure the reader. Would it make any sense to plot the cross-correlation between speech and filtered components?

l118: Tell us more about how the frequency shifter works. Do you really mean ‘transformer’?

l119: this paragraph is unclear. Not clear what is meant by ‘generate as well as measure’. Perhaps ‘generate’ --> ‘elicit’? l124 seems to imply that w_{2m-n}(t) is a distortion product waveform, but it seems instead that it is a harmonic of rank 2m-n that you synthesize so as to be able to reveal the distortion product by cross-correlation. Why that is a good idea is a bit mysterious. Elaborate!

l125 and elsewhere: consider replacing ‘sideband’ by ‘partial’ or ‘distortion product’. Sideband is widely (mis)used in the DPOAE literature, but its real meaning is the spectral consequence of modulating a sinusoidal carrier, which doesn’t fit this situation.

l128: Again, it is not clear to me why it is important to consider the relation between w_{2m-n}(t) and the DPOAE (phase and delay). What does it tell us?

Eq1: actual calculations are on samples. Would it be possible to convert Eq to involve sampled quantities (e.g. \sum instead of \int) without making things less clear?

l135: Why must C(0) be 1? It isn’t in Fig. 2 C & D (if those plots represent crosscorrelation). Why would the imaginary part of C be zero at \tau=0?

l136: ‘amplitude’ --> ‘magnitude’? It would help if you gave a few more details.

l143: ‘best’ --> ‘easiest’?

l153: The boundary between resolved and unresolved depends on the criterion and is not the same in every study. It’s not all or nothing.

l174: ‘in quiet’ --> ‘in isolation’

l178: why 12?

l182: why 3? Reorganize the description.

l196: I don’t see how this is comparable to speech OAEs. What phase do you attribute to the 800 Hz tone?

l200: ‘peak amplitude’ --> ‘peak correlation’ (or ‘peak correlation magnitude’)?

l204, ‘To...’: perhaps move to the description of the equipment

l210: 95% of 95% of the noise?

l223, ‘amplitude’: do you mean peak value of correlation? If so, use that term.

l233: what are the ‘three corresponding stimuli’?

Fig2 Caption: describe each panel before telling us what is worth noting. The meaning of ‘saturated’ is obscure. Omit or define in the Methods.

l270: it’s unclear what to expect from placing the probe outside the ear canal, since you give no details in Methods. Transducer and microphone are coupled even if the probe outside the ear canal?

l280: units?

Fig3: C: axes have same label. DPOAE is a phenomenon, not a quantity. Units? ‘dB SPL’-->’dB SPL / Hz’ presumably. Caption: describe each panel before telling us what is worth noting.

l288: Is power density the right concept? For a line spectrum the density is infinite at each line, which means in practice that values depend on the size of the analysis window.

l308: then-->them

l311: Do speech-OAEs differ between with speech vs without speech in the other ear?

l357: this can’t be true as stated because phase is proportional to frequency for any sinusoid. I believe what you mean to say is that phase effects due to frequency-dependent phase variations affect a short latency response less than a long latency response. However, that too is incorrect: what counts is not the latency, but the duration of the impulse response, which you don’t discuss here. Please rewrite.

l362: why didn’t you use the same harmonics for male and female? Is there a reason to believe that higher frequencies would significantly reduce DPOAE amplitudes?

l371: Strictly speaking, a discussion of the difference between a condition that yields a significant effect and one that does not is not founded. You need to test whether the between-condition difference of effect size is significant (interaction). Perhaps formulate as: "We can speculate that the lack of significant effects for male voices might reflect poor resolvability of the target harmonics...’

l373: what does "attentional modulation of the resolved harmonics" mean?

l378, ‘the cochlea appears’: is this based on the present data or from the literature?

l387: what does ‘intermodal attention’ mean?

l389: in what way is this related to ‘temporal fine structure’? (or perhaps: in what way could it not be?) Reformulate to make the reasoning explicit.

l406: ‘related’ --> ‘relates’ or ‘is related’ ?

l410: ‘reduced’ --> ‘small’. ‘difference across conditions’ --> ‘lack of effect for male’

I’m a bit concerned by your statement that significance is ‘marginal’. On the one hand, this degree of self-scepticism is refreshing and commendable. On the other, it does seem to invoke the spectre of yet another non-replicable effect. Ideally you should recruit a new cohort of subjects and replicate, but that is perhaps difficult given the pandemic. My feeling is that the risk is worth taking if the methodology is solid and well explained.

References are incomplete (they lack volume and page numbers). Spell out all words unless perhaps standard journal abbreviations. Check journal style guide.

Reviewer 2:

The contents of the manuscript can be divided into two parts. The first part is the presentation of a novel method of recording cochlear distortion using analogues of harmonics of the fundamental frequency of human voice. The second part includes the use of this method to evaluate attentional modulation of cochlear distortion.

The premise of the paper is a solution to the heretofore unresolved contribution of the cochlea to understanding speech in the presence of background or distracting signals. Many have used otoacoustic emissions in general and efferent modulation of otoacoustic emission amplitude to examine whether central modulation of cochlear amplification is part of the solution enabling understanding of speech in the presence of distracting signals. The authors claim that previous work has been ambiguous because cochlear emissions have been recorded using signals that do not resemble human speech.

The results suggest that cochlear distortion can be recorded using the novel method presented in the manuscript, that this distortion is correlated in magnitude to that measured using the traditional steady sinusoidal signals, and attending to the female but not the male voice leads to an increase in the amplitude of cochlear distortion. This last finding is explained by suggesting that the harmonics of the female voice perhaps engender a modulation effect as they are in the range of resolved harmonics, whereas the harmonics used for the male voice were not.

The authors do caution the reader about their limited sample size. Unfortunately this is not enough in the opinion of this reviewer. Measures of efferent modulation of cochlear distortion are notoriously variable. For instance, Oxenham et al. have recently demonstrated that the direction of an effect observed in one sample of ears can be completely reversed in the next sample. At the very least, some attempt to demonstrate stability of these results even within this subject pool would be needed. For example, could some form of bootstrapping be used to establish reproducibility of the results?

The claim that measuring cochlear distortion using a speech-like signal leads to substantially different outcomes can only be claimed if a direct comparison with results obtained using the traditional methods was presented within subjects.

Finally, the claim that the lack of attentional effects in the male voice was because of unresolved harmonics could be easily verified by lowering the order of harmonics and repeating the experiment.

Overall it is a well written manuscript with the novel method presented adding value to the literature. If the authors are willing to recast the manuscript to focus on the methods and attenuate the claims about attentional modulation of cochlear distortion, the paper would be accurate and useful.
